# Highlights on the Effects of Non-Coding RNAs in the Osteonecrosis of the Jaw

**DOI:** 10.3390/ijms25031598

**Published:** 2024-01-27

**Authors:** Santino Caserta, Fabio Stagno, Sebastiano Gangemi, Alessandro Allegra

**Affiliations:** 1 Hematology Unit, Department of Human Pathology in Adulthood and Childhood “Gaetano Barresi”, University of Messina, Via Consolare Valeria, 98125 Messina, Italy; santino.caserta@polime.it (S.C.); aallegra@unime.it (A.A.); 2 Allergy and Clinical Immunology Unit, Department of Clinical and Experimental Medicine, University of Messina, Via Consolare Valeria, 98125 Messina, Italy; gangemis@unime.it

**Keywords:** osteonecrosis of the jaw, epigenetics, non-coding RNA, miRNA, circularRNA, long-non codingRNA, osteoclast, neoangiogenesis, inflammation, multiple myeloma

## Abstract

Osteonecrosis of the jaw is the progressive loss and destruction of bone affecting the maxilla or mandible in patients treated with antiresorptive and antiangiogenic agents without receiving prior radiation therapy. The pathogenesis involves the inflammatory pathway of receptor activator of nuclear factor NF-kB ligand and the macrophage colony-stimulating factor, essential for osteoclast precursors survival and proliferation and acting through its receptor c-Fms. Evidence has shown the role of non-coding RNAs in the pathogenesis of osteonecrosis of the jaw and this finding might be useful in diagnosis since these small RNAs could be considered as biomarkers of apoptotic activity in bone. Interestingly, it has been proved that miR-29 and miR-31-5p, acting on specific targets such as CALCR and RhoA, promote programmed-cell death and consequently the necrosis of bone tissue. Specific long non-coding RNAs, instead, have been detected both at reduced levels in patients with multiple myeloma and osteonecrosis, and associated with suppression of osteoblast differentiation, with consequences in the progression of mandible lesions. Among non-coding genic material, circular RNAs have the capability to modify the expression of specific mRNAs responsible for the inhibition of bisphosphonates activity on osteoclastogenesis.

## 1. Osteonecrosis of the Jaw

The Italian Society of Oral Pathology Medicine (SIPMO) and the Italian Society for Maxillofacial Surgery (SICMF) define osteonecrosis of the jaw (ONJ) as the adverse drug reaction characterized by the progressive loss and destruction of bone that affects the maxilla or mandible in patients treated with antiresorptive and antiangiogenic agents without having received prior radiation therapy [1,2]. Regarding the clinical features, earlier research has revealed that most patients at risk are female, have an average age of 65.3 years, and that the mandible is the most often affected jawbone [3,4]. ONJ consists of three stages: (i) Stage 0 does not involve a fistula or exposed bone, it is considered a possible precursor to the disease; odontalgia, maxillary sinus discomfort, jaw pain that may extend to the temporomandibular joint region, and altered feeling in the maxilla and mandibular areas are among the symptoms; there could be movement of a tooth and intraoral or extraoral edema that isn’t connected to periodontal disease, such as the absence of formation of new bone in the extraction socket, alterations of the structure of bone, osteosclerosis regions, and thickening of the periodontal ligament [5]; (ii) Stage 1 involves a fistula or exposed necrotic bone without any signs of inflammation; radiographic images may be limited to the alveolar bone region and resemble those of Stage 0; (iii) in Stage 2, patients exhibit symptoms such as an exposed necrotic bone or a fistula, along with signs of infection and inflammation; (iv) Stage 3 is characterized by an exposed and necrotic bone or fistulae that probes the bone, along with infection and pathological fracture, extraoral fistula, oral–antral or oral–nasal communication, osteolysis extending to the inferior border of the mandible or sinus floor, or exposed necrotic bone extending beyond the region of the alveolar bone as complications [6,7,8].

### 1.1. Pathogenesis

#### 1.1.1. Inflammatory and Growth Pathways

Three signaling pathways primarily regulate osteoclast differentiation: macrophage colony-stimulating factor (M-CSF), receptor activator of nuclear factor NF-κB ligand (RANKL), and immune receptor tyrosine-based activation motif (ITAM) [9,10,11]. M-CSF is essential for osteoclast precursors (OCPs) to survive and proliferate; in fact, it signals the cell via its receptor c-Fms, which in turn triggers phosphoinositide 3-kinase (PI-3K)/Akt and extracellular signal-regulated kinase (ERK) via Growth factor receptor bound 2 (Grb2). Purine-rich binding protein 1 and Microphthalmia transcription factor (Mitf) are two examples of transcription factors that stimulate the differentiation of hematopoietic stem cells into OCPs. In the Erythroblast transcription factor (ETS) family, Spi1 proto-oncogene (PU.1) is unique to hematopoiesis and it has been shown that when mice with PU.1 deletion have no OCPs at all, osteopetrosis occurs. In detail, hematopoietic stem cells are stimulated by PU.1 to express the Colony-stimulating receptor (CSF1R) during the process of development into the monocyte/macrophage lineage; CSF1R stimulates the transcription factor c-FOS, which in turn causes RANKL to be expressed, and PU.1 modifies the transcription of the RANK gene in concert with other transcription factors. Moreover, in the process of osteoclastogenesis, activator protein 1 (AP-1) is essential. The Fra, Fos, Ju, and MITF transcription factors make up the AP-1 transcription factor complexes. Moreover, MITF is an essential transcription factor involved in the last phases of osteoclastogenesis and it is phosphorylated by a conserved mitogen-activated protein kinase (MAPK) consensus site. Next, it increases macrophage survival by promoting BCL-2 expression. Furthermore, MITF and PU.1, by attaching themselves to the RANK promoter’s sites, jointly boost the activity of the RANK promoter by three and two times, respectively, and six times. On the other hand, RANKL dramatically upregulates Mitf-E levels that, together with PU.1, are involved in the induction of osteoclast-specific genes. Genes responsible for the fusion of mononuclear osteoclast precursors, such as dendritic cell-specific transmembrane protein (DC-STAMP), and genes regulating the resorption capacity of multinucleated osteoclasts, such as cathepsin K, chloride channel 7, matrix metalloprotein 9, and calcitonin receptor, are expressed when RANKL–RANK signaling is activated. The NF-κB family of transcription factors together with cytokines, such as interleukin 6 (IL-6), interleukin 1 (IL-1), Tumor Necrosis Factor-alpha (TNF-α), granulocyte-macrophage colony-stimulating factor (GM-CSF), RANKL and other growth factors expressed, act with the aim to activate PI-3K [12,13,14]. MAPK kinase 6 phosphorylation, finally, leads to the activation of protein kinase p38, which in turn activates MITF. Osteoclast-associated receptor (OSCAR), Cathepsin K, tartrate-resistant acid phosphatase (TRAP), calcitonin receptor, and β3 integrin are examples of osteoclast-specific genes that are regulated by Nuclear factor of activated T cells 1 (NFATc1). Multiple immunoreceptors that are activated by ITAM-dependent costimulatory signals work in concert with RANKL; as a result, co-stimulatory signals for RANK can be recognized as ITAM-mediated signals [15,16].

#### 1.1.2. Five Pathophysiology Theories

**Figure 1:**
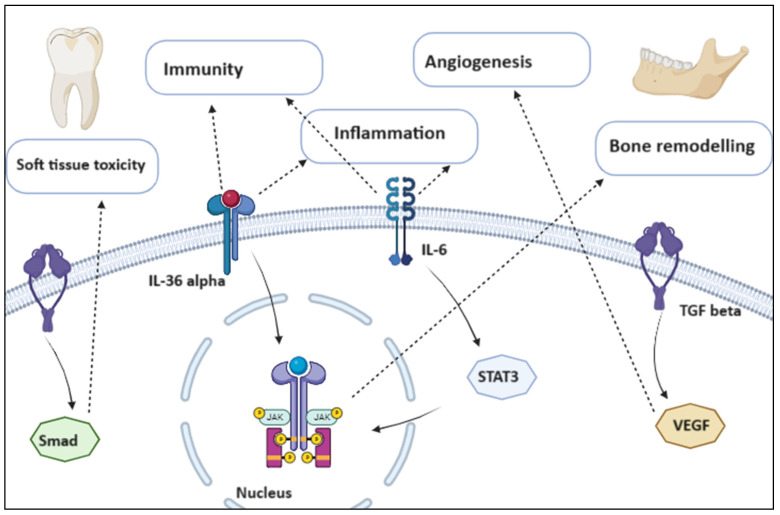
Pathogenetic conditions associated with osteonecrosis of the jaw. Created with BioRender.com (accessed on 19 November 2023).

In response to the paradox of ONJ pathophysiology, different explanations have been proposed: inhibition of osteoclastic bone resorption and remodeling, inhibition of angiogenesis, inflammation and infection, immunological dysfunction, and soft tissue toxicity. As stated by the theory of altered bone remodeling, since the target cell for treating osteoporosis and metastatic bone cancer is the osteoclast and drugs like bisphosphonates target them inhibiting their function, the basic theory behind ONJ is altered bone remodeling. Angiogenesis inhibition is an additional mechanism; in fact, when combined with vascular dysfunction, zoledronic acid’s direct inhibition of angiogenesis may result in the development of the disease. Angiogenesis inhibition prevents human endothelial cells from adhering to each other and migrating, hence preventing tumor invasion and metastases. Another theory, based on initial findings on animals, recognizes the central role of inflammation and infection, since after the sick teeth were extracted from animals receiving antiresorptive medication, the animals displayed poor healing, including mucosal abnormalities, significant inflammatory infiltrates in the sockets, bone exposure and osteonecrosis zones [6] (Figure 1).

#### 1.1.3. Gamma-Delta T Cell Impairment

The oral microbiota composition of patients with ONJ, patients using bisphosphonates without osteonecrosis of the jaw, and healthy controls was examined in a clinical investigation and the results showed that oral bacterial community did not differ between the groups. Rather, there was a robust correlation between the systemic leukocyte activity and the composition of their oral microbiota. In fact, its composition has been observed to be correlated with peripheral blood leukocytes’ expression of the RANK, TNF-alpha, and aryl hydrocarbon receptor genes [17,18,19] linked to stress and immunological resilience. Immune system dysfunction theory compared human and rat ONJ necrotic bone samples to healthy patients and non-ONJ locations and revealed significant differences in the quantity and distribution of T-cells. It has been shown that gamma-delta T cells have a well-established function in antimicrobial immunity, and they are distinguished from alpha-beta T cells by their recognition of antigens. Gamma-delta T cells are innate lymphocytes crucial for bone regeneration, whereas alpha-beta T cells have been shown to promote bone loss. Interleukin-17A (IL-17A), which is produced by these T cells, aids in the growth of new bone and the healing of fractures. Studies on patients with osteonecrosis receiving bisphosphonate medication revealed a notable decrease in gamma-delta T cells; moreover, the immunological integrity was further weakened by underlying diseases. The last proposed theory is that of soft tissue toxicity, since it has been demonstrated that bisphosphonates are harmful to oral keratinocytes and fibroblasts, and they have adverse effects on the viability, apoptosis, proliferation, and migration of cells. In fact, keratinocytes were prevented from finishing their cell cycles during cell proliferation, and fibroblasts underwent apoptosis. Furthermore, in ONJ human biopsy tissue, it was found that delayed periodontal repair was associated with altered transforming growth factor beta 1 (TGFβ1) signaling [6].

## 2. Evidence from Non-Coding RNAs in the Osteonecrosis of the Jaw

The aim of our work is to explore the involved pathways of non-coding RNAs and evaluate their possible role in the pathogenesis, progression, and prognosis of osteonecrosis of the jaw, based on the most recent scientific literature. In this view, a search on PubMed was performed using the keywords “Osteonecrosis of the jaw”, “non-coding RNAs”, “miRNAs and osteonecrosis”, “Long non-coding RNAs and osteonecrosis”, and “Epigenetics” in a period from 2008 to 2023.

microRNAs (miRNAs) are a class of non-coding RNAs of about 22 nucleotides that have been substantially conserved throughout evolution. The precursor of miRNAs is a lengthy ribonucleic acid molecule known as pri-miRNA, which is capped and polyadenylated in the nucleus by polymerase II. Subsequently, the pre-miRNA, which is composed of roughly 70 nucleotides, is processed from Drosha proteins and DiGeorge syndrome critical region 8 (DGCR8). Following its passage through the exportin 5-RAs-related nuclear protein (RAN) complex to the cytoplasm, this molecule undergoes endonuclease-mediated maturation into miRNA. Its mechanism of action involves attaching to the 3′ untranslated sections of mRNA and preventing its translation or stimulating protein degradation. It can also be released into vesicles termed exosomes in the extracellular microenvironment or circulate linked to lipoproteins and argonaut proteins [5].

RNA molecules known as long non-coding RNAs (lncRNAs) control the transcriptional, post-transcriptional, and translational stages of gene expression. They are unable to code and are longer than 200 nucleotides [20]. Aberrant lncRNA expression has been connected to a number of human diseases, which emphasizes the need for a deeper understanding of disease etiology to inform advancements in diagnostic, prognostic, and therapeutic approaches. lncRNAs are categorized as sense, antisense, bidirectional, intronic, and intergenic based on the transcription locations in relation to identified protein-coding genes. They are essential for the transcriptional and post-transcriptional control of genes and regulate basic cellular processes and pathways through their involvement in chromatin remodeling, epigenetic changes, and subcellular compartmentalization [21].

Nuclear macromolecules known as circular RNAs (circRNAs) are made up of ribonucleotide sequences that lack polyadenylated tails and have a circular shape. They are categorized as Housekeeping ncRNAs, albeit some of them can code for certain proteins. In total, 1976 tiny segments of circular, non-coding material—known as splicing errors—were extracted from viroids infecting plants, and these segments were then used to detect eukaryotic cells, such as the mitochondrial RNAs of some yeasts, and the hepatotropic virus δ [20].

## 3. miRNAs

A class of non-coding RNAs of about 22 nucleotides that have been substantially conserved throughout evolution are known as microRNAs (miRNAs) [5]. Through their binding to different mRNA targets, miRNAs regulate the expression of genes post-transcriptionally and they control the differentiation of osteoclasts and play relevant regulatory roles in osteogenic differentiation, including osteogenesis and cartilage formation. As post-transcriptional regulators of gene expression, miRNAs are essential for the healthy development of bones [22,23,24]. Single nucleotide polymorphisms (SNPs) associated with microRNA may also influence the skeletal phenotype. New data points to the involvement of miRNAs in a variety of physiologic and pathological processes, including osteoclast development, proliferation, programmed cell death, cytoskeleton creation, and bone resorption. MiRNA microarray investigation revealed that 44 miRNAs were downregulated and 49 miRNAs were upregulated throughout the early, middle, and late phases of murine osteoclastogenesis (Table 1) [15]; miR-29, miR-31-5p, hsa-miR-422a, hsa-miR-148a-3p, miR-183-5p, miR-214-3p, and miR-9718 were found to be expressed in a way that promoted osteoclastogenesis, while miR-7b-5p, miR-26a-5p, miR-34a-5p, miR-124-3p, miR-125a-5p, miR-146a-5p, miR-218-5p, and miR-503-5p were found to be expressed in a way that inhibited osteoclastogenesis. Furthermore, three miRNAs—miR-21-5p, hsa-miR-133a-3p, and miR-223-3p—were demonstrated to have both promotion and inhibitory effects [6]. In a recent study, patients with multiple myeloma and with or without ONJ had their miRNA levels examined and it was demonstrated that miRNAs miR-16-1, miR-21, miR-23a, miR-28, miR-101-1, miR-124-1, miR-129, miR-139, miR-145, miR-149, miR-202, miR-221, miR-424, and miR-520 were considerably overexpressed in ONJ patients compared to controls. In detail, it was shown which miR-23a primarily targets specific genes such as osteoclast stimulating factor 1 (OSTF1), secreted protein acidic and rich in cysteine (SPARC) and SPOCK1, low-density lipoprotein receptor-related protein 5 (LRP5), which is closely linked to steroid-associated femoral head necrosis, interleukins, tumor necrosis factor, TNF-related targets, and Fos proto-oncogene (FOS). Since miR-23a is one of the highly abundant miRNAs in human umbilical vein endothelial cells, selective expression of this miRNA in cells has been demonstrated in vivo in microvascular endothelial cells, suggesting a possible interplay with angiogenesis.

The microRNA family’s miR-23 has been linked to osteoporosis and the control of osteoblast differentiation, targeting the phosphatase and tensin (PTEN) gene to regulate osteoclast differentiation [25,26,27,28]. Moreover, miR-21 affects the expression of osteoprotegerin (OPG) and receptor activator of nuclear factor kappa-B ligand (RANKL) to suppress OPG and to improve osteogenic differentiation by targeting Suppressor of Mothers against Decapentaplegic7 (Smad7), sprouty RTK signaling antagonist 1 (Spry1), and periodontal-ligament-associated protein-1 (PLAP1). It is associated with the pathophysiology of particle-induced osteolysis, and inhibition of osteoclastogenesis resulting from lowering of miR-21. MiR-21 variation in pro-osteoclastogenesis is consistent with the upregulation of circulating miR-21 during ONJ progression [29,30]. The primary targets of miR-145 instead include interleukin (IL) and genes related to bone homeostasis, such as osteomodulin, cwcv, and kazal-like domains proteoglycan 2, SPARC/osteonectin, and secreted protein acidic and rich in cysteine [6,31,32]. Additionally, miR-21 showed a favorable link with the pathophysiology of particle-induced osteolysis, and its knockdown-restricted osteoclastogenesis. It has been established that miR-145 is upregulated not only during osteoblast differentiation but also in relation to monocyte-related osteoclastogenesis that is triggered by RANKL. On the other hand, steroid-induced avascular necrosis of the femoral head was prevented by silencing miR-145. According to recent data, ONJ participants had higher blood levels of miR-21 and miR-23a and lower serum levels of miR-145, all of which were consistent with the cellular roles described by earlier studies [33]. Zoledronic acid (ZOL) has the ability to alter the expression of miRNAs; in fact, it has been shown that ZOL therapy at a low dose significantly altered 54miRNAs’ expression. After a 24 h treatment period, nine upregulated and twelve downregulated miRNAs were identified. Additionally, ZOL decreased the expression of 22 miRNAs while increasing the expression of 11 miRNAs. MiRNA data analysis revealed the involvement of differentially expressed miRNAs in actin cytoskeleton control and JAK-STAT, PI3K/Akt, Wnt, MAPK, and TGF-β signaling pathways. These findings appear to support the hypothesis that dysregulation of miRNAs plays a part in the development of osteonecrosis of the jaw. mir-23a can help in regulating the cell cycle and it is selectively expressed in vivo by microvascular endothelial cells. It is one of the highly expressed miRNAs in human umbilical vein endothelial cells, suggesting that it may play a role in angiogenesis. miR-520e gene is located on 19q13.42, and mir-520e directly targets NF-κB-inducing kinase (NIK). As a member of the mitogen-activated protein kinase family, NIK plays a part in regulating the mechanisms involved in OC mitochondrial biogenesis and differentiation. Furthermore, although lymphocytes seem to be more significant numerically, it is still possible that monocytes contribute to the dysregulation of miRNAs in ONJ patients and it has been suggested that bisphosphonates promote osteonecrosis of the jaws by interfering with the growth and function of monocytes. Bisphosphonate injection has been shown to alter the monocyte-mediated immune response, resulting in a drop in CD14 peripheral blood monocyte (PBMC) populations and an increase in CD14+ PBMC. In conclusion, the start of ONJ may be related to a changed microRNA expression profile following ZOL therapy; by focusing on these miRNAs, a novel therapeutic approach for the avoidance or management of ONJ may be developed [30].

### 3.1. miRNAs as Biomarkers and Therapeutic Targets of Osteoclasts Activity

The expression of the miR-29 (a/b/c) family, a positive regulator of osteoclast formation, was elevated during osteoclastogenesis in vitro cell culture. This fact agrees with the expression of the osteoclast markers cathepsin K and TRAP [34,35]. In contrast, the decreased expression of TRAP and Cathepsin K was consistent with the overexpression of miR-218-5p, a negative regulator of osteoclastogenesis [36]. Since osteoclast activity has been measured using plasma TRAP5b expression, more research is necessary to confirm the potential of miR-29 and miR-218-5p as novel biomarkers for osteoclast activity. Yao and colleagues successfully delivered miR-146a into human PBMCs using a new miRNA delivery system based on bacteriophage MS2 virus-like particles (MS2 VLPs) and they later showed the inhibitory role of miR-146a-5p in osteoclastogenesis [37]. Furthermore, strong inhibitory effects of miR-148a-3p and miR-503-5p are seen in osteoclast differentiation [38,39,40]. After utilizing a specific antagomir to mute miR-503-5p expression, animals after ovariectomy showed an increased production of RANK protein, enhanced bone resorption, and decreased bone mass, while agomir-503 showed the opposite effects. When creating miRNA-based therapies, it would be beneficial to preserve osteoblasts’ regular functions, even if this means merely blocking osteoclasts’ ability to resorb bone without changing the overall quantity of osteoclasts. Consequently, it appears that miR-31-5p will be a perfect target because it only enhances osteoclast function by blocking Ras homolog family member A (RhoA), and RhoA is a small GTPase that is essential for actin ring formation [15].

### 3.2. mir-149-5p Modulates the Rap1a/Rap1b/VEGFR2 Pathway

Zoledronic acid promoted the transcription of miR-149-5p by molecularly activating NF-κB signaling and inducing p65 nuclear translocation in macrophages. Then, Traf6 3′-UTR was the direct target of overexpressed miR-149-5p, which inhibited osteoclast differentiation. It was discovered that bone marrow-derived endovesicles (EVs) loaded with miR-149-5p shuttled to EVs, whereupon they inhibited the biological functions of ECs through the Rap1a/Rap1b/VEGFR2 pathway. Serum samples from ONJ mice and patients both have high levels of miR-149-5p; thus, blood sample levels of mature miR-149-5p transcripts could be used as a possible ONJ diagnostic sign [41]. At a therapeutic level, ONJ was dramatically reduced in the mouse model when miR-149-5p expression was decreased, indicating that miR-149-5p may be a useful target for the treatment of osteonecrosis. During skeletal development and bone regeneration, angiogenesis—especially of type H vessels—and osteogenesis are closely related processes [41,42]; in fact, blood arteries are essential for bone regeneration during the bone-healing processes, in addition to supplying bone tissues with the nutrition, oxygen, and growth substances they require [43,44]. Type H vessels, which have a distinct shape and geographic distribution pattern, have also been recently discovered by Yan and colleagues in alveolar bone [22]. These unique vessel subtypes originate from the Sharpey’s fiber-lining cancellous alveolus plate. In ONJ mice, scientists observed a substantial decrease in the number of CD31hiEMCNhi type H vessels and a decrease in OSX+ perivascular osteoprogenitors. According to Xiong and colleagues, salt-inducible kinases 2 and 3 are the target of exosome miRNA-5106, which is generated from M2 macrophages and drives bone mesenchymal stem cells toward an osteoblastic destiny [45]. Furthermore, in a myocardial infarction milieu, M1-like macrophage-derived EVs have been shown to restrict angiogenesis and worsen cardiac dysfunction [11]. Due to the high expression level of miR-149-5p, it was discovered that ZOL-treated EVs transport regulatory components that restrict proliferation, migration, and tube formation [30]. In addition, it has been shown that the overexpression of miR-149-5p directly inhibited the expression of Traf6, which is connected to the RANK/RANKL pathway, hence modulating osteoclast development. Since the expression of miR-149-5p was shown to influence macrophage polarization, it could be stated that macrophage-derived EVs play a crucial role in controlling the development of skeletal angiogenesis in response to ZOL administration: EVs equipped with miR-149-5p have the ability to suppress osteoclastic development; this implies that targeting miR-149-5p could be a potential therapeutic approach in ONJ [42].

## 4. Long Non-Coding RNAs

A collection of noncoding RNAs longer than 200 base pairs is known as long noncoding (lnc) RNAs. Since most lncRNAs include a poly-A tail, their biogenesis is comparable to that of mRNA and protein-coding RNAs; nevertheless, they are not able to be translated into proteins [43]. Only a small portion of the 15.778 human lncRNAs that have been identified to date have been characterized. Enhancer RNAs, intergenic transcripts, and snoRNA hosts are examples of lncRNAs. They are found in nearly all cell types and are key players in many cellular processes, including nuclear-cytoplasmic transit, cell cycle progression, cellular architecture, transcriptional and posttranscriptional regulation, and proliferation of cells. Additionally, they influence how gene expression is regulated epigenetically [44,45,46,47]. Different modes of action exist for lncRNAs, since they can facilitate the formation of a quaternary structure for proteins, fold into a tertiary structure and control the stability of mRNAs, alter the efficiency of target mRNA translation, and determine increased mRNA expression in order to regulate the expression of genes at the posttranscriptional level. Over 3000 lncRNAs were found to be dysregulated in patients with multiple myeloma and 176 lncRNAs were found to be prognosis biomarkers. Furthermore, changes to lncRNAs may play a significant role in the development and course of the illness. lncRNA KIAA0495 was shown to have a progressive downregulation from MGUS to symptomatic MM after healthy controls. Lastly, lncRNAs may be crucial for bone metabolism and possibly for the development of MM bone disease. The role of long noncoding RNAs during osteogenic lineage commitment or osteocyte terminal differentiation has been assessed in recent experiments: lncRNA-1, for instance, showed increased expression during osteogenesis. In contrast to MM patients without ONJ and healthy controls, scientists found that MM patients with ONJ had a distinct lncRNA profile. Differentiation Antagonizing Nonprotein Coding RNA (DANCR) inhibits the Wnt/β-catenin pathway and blocks the p38MAPK pathway, which in turn reduces osteogenic differentiation [48,49,50]; additionally, the transcription factor FOXO1 is expressed less when this lncRNA is expressed, which raises osteoblast differentiation and lowers their proliferation [45,46]. Downregulation of DANCR was shown to be essential for osteogenesis in a study examining DANCR expression in human periodontal ligament stem cells. According to these findings, it can be stated that MM patients with ONJ had lower expression levels of DANCR [51].

### 4.1. lncRNAs Modulate Osteogenic Proliferation and Differentiation

Reduced levels of lncRNA in MM patients with ONJ may be associated with suppression of osteoblast differentiation, which impacts the mandibles during lesion progression. A comparable significance could be associated with the downregulation of Metastasis-Associated Lung Adenocarcinoma Transcript 1 (MALAT1). It regulates integrin concentrations, including ITGB1, which is important for osteoclast formation and cytoskeletal structure. By interacting with Dlx transcription factors, MALAT1 inhibits miR-124, which in turn negatively influences osteogenic differentiation and bone production. In comparison to both controls and MM patients, data showed a considerable down-expression of MALAT1, which is likely due to enhanced osteoclast generation brought on by bone lesions. Additionally, the overexpression of several lncRNAs may negatively impact osteogenesis through various methods. Osteogenic differentiation can be inhibited by HOX Transcript Antisense RNA (HOTAIR). When comparing patients with osteonecrosis of the femoral head not due to trauma to samples with osteoarthritis, it was shown that the expression of HOTAIR was higher in the former group. Si-HOTAIR boosted the concentration of osteogenic differentiation indicators, including COL1A1 and RUNX2 mRNA levels. Furthermore, because HOTAIR is mechanoresponsive, it might contribute to mechanically regulated calcification. In comparison to both MM patients and controls, HOTAIR showed increased expression in MM patients with ONJ; these outcomes were consistent with the observations reported in nontraumatic femur osteonecrosis, suggesting that this lncRNA may have a deleterious effect on osteogenic proliferation and differentiation. H19, a 2.3 kb long non-coding RNA, functions as a “sponge” to lessen the effects of microRNAs that alter the production of pro-osteogenic proteins, such as the Wnt/β-catenin pathway and its target genes. It can also regulate several elements with a regulatory effect on osteogenesis. Research findings showed that H19 expression levels were higher in MM patients than in controls, which may have a detrimental effect on osteogenesis [51].

### 4.2. lncRNAs Inhibit Bone Neoangiogenesis

An alternative pattern of lncRNA production may also be crucial, and overexpression of lncRNAs like Jumonji or Maternally Expressed 3 (MEG3) may lead to a significant reduction in vascularization in ONJ patients. While MEG3-knockout animals showed increased expression of vascular endothelial growth factor (VEGF) pathway genes and microvessel density, the MEG3 lncRNA gene can alter the expression of genes that promote angiogenesis. In comparison to controls and MM, scientific findings indicate that MEG3 is increased in MM patients with ONJ. It is possible that its effect on angiogenesis contributes to the development of the characteristic avascular necrosis in ONJ. The meaning of Jumonji C’s overexpression might be similar since it can decrease angiogenesis [45], and its elevation may play a role in the development of ONJ-specific microinfarcts. Similarly, the overexpression of additional lncRNAs may influence osteogenesis and angiogenesis in a favorable way. Endothelial cells express the lncRNA n342419 also termed MANTIS, and a decrease in MANTIS expression modifies endothelial sprouting and diminishes endothelial migration [46]. According to recent research [47], bisphosphonates significantly impair angiogenesis, revascularization, and microvessel sprouting. When MM patients with ONJ are compared to controls or other MM patients, MANTIS is increased. It is possible that this increase results from tissue attempting to promote bone regrowth. By binding to miR-153-3p, HIF1A-AS2 lncRNA moderates the rise in HIF-1α while concurrently stimulating angiogenesis [51].

## 5. circularRNAs

**Figure 2:**
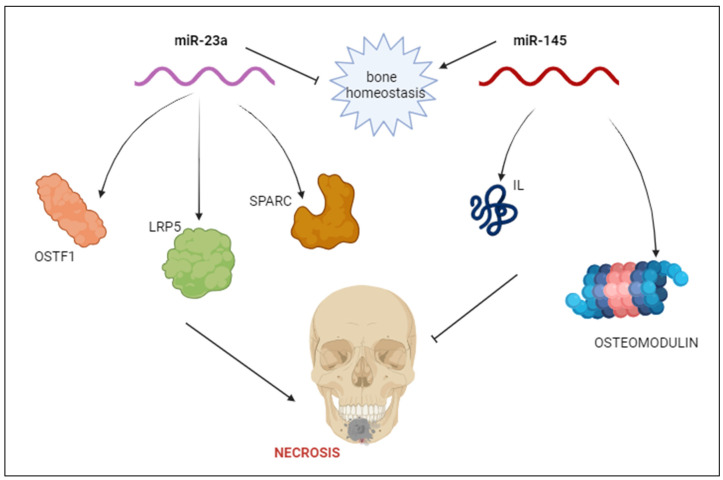
Patients with ONJ showed higher blood levels of miR-23a and lower serum levels of miR-145: miR-23a targets specific genes such as osteoclast stimulating factor 1 (OSTF1), secreted protein acidic and rich in cysteine (SPARC) and SPOCK1, low-density lipoprotein receptor-related protein 5 (LRP5), while miR-145 acts on interleukin (IL) and genes related to bone homeostasis, such as osteomodulin, and secreted protein acidic and rich in cysteine.

It has recently been discovered that circRNAs function as “sponges” of miRNAs, indirectly regulating the genes that miRNAs target and altering several biological processes [52]. To current knowledge, few investigations have examined circRNAs as a potential ONJ biomarker. Standardizing the diagnosis of ONJ might be possible by a valuable biomarker, which could be also used to evaluate the risk in patients undergoing bisphosphonates treatment [52,53,54,55]. Numerous studies have hypothesized that circRNAs may sequester miRNAs to modify mRNA expression [56] and some of them, via their effects on ONJ-related miRNAs, are essential in RANKL-induced RAW264.7 cells treated with bisphosphonates [57] (Figure 2).

### mmu_circ_0001066 Attenuates the Inhibitory Action of Bisphosphonates on Osteoclastogenesis

It was determined that 79 circRNAs might potentially sequester miR-16, miR-221, miR-23a, and miR-145a. For additional verification, scientists chose seven circRNAs (mmu_circ_0001162, mmu_circ_0001144, mmu_circ_0001066, mmu_circ_0000296, mmu_circ_0001599, mmu_circ_0001219, and mmu_circ_0000377) that interacted with two or more miRNAs (miR-16, miR-221, miR-23a, and miR-145a). The expression of the seven circRNAs in RANKL-induced RAW264.7 cells treated with 10-6 M ZOL was examined in order to test the hypothesis and it was demonstrated that, in the ZOL-treated group as compared to the control one, the expression of mmu_circ_0001066 was lowered to 0.2-fold, whereas the expression of mmu_circ_0001162, mmu_circ_0001144, mmu_circ_0000296, mmu_circ_0001599, and mmu_circ_0000377 did not change significantly [12]. Furthermore, mmu_circ_0001219 data was not obtained for the qRT-PCR study. When considered together, jaw osteonecrosis-related miRNAs and mmu_circ_0001066’s expression showed a negative correlation. Bioinformatics findings theoretically allowed for the selection of mmu_circ_0001066 as the study’s final candidate circRNA and overexpressed mmu_circ_0001066 by transfecting the mmu_circ_0001066 plasmid into RAW264 to investigate the impact of it on the BP-induced suppression of osteoclast development in more detail. To validate the effectiveness of the mmu_circ_0001066 transfection, cells and qRT-PCR were utilized, then TRAP staining and activity data were examined with the aim of evaluating mmu_circ_0001066’s function [58]. Comparing the mmu_circ_0001066 group to control groups, there was a significant increase in the number of TRAP-positive multinuclear cells, and mmu_circ_0001066 overexpression raised the TRAP activity to four times. Additionally, to confirm the impact of this circular RNA on osteoclastogenesis, Western blots were employed and MMP-9 and cathepsin-K, two proteins linked to osteoclastogenesis, were found to be increased when mmu_circ_0001066 was overexpressed. An outward sign of mature osteoclast development and bone resorption is a well-polarized F-actin ring. Therefore, to find out how mmu_circ_0001066 affected BP-inhibited osteoclastogenesis, F-actin ring staining was performed. According to the findings, mmu_circ_0001066 facilitated the development of an F-actin ring encircling many nuclei. Furthermore, researchers evaluated the impact of the mmu_circ_0001066 inhibitor on RANKL-induced osteoclastogenesis in the absence of BP therapy. The mmu_circ_0001066 inhibitor greatly reduced osteoclast differentiation, as demonstrated by TRAP staining and activity. These findings led to the conclusion that mmu_circ_0001066 overexpression prevented RAW264.7 cells’ osteoclast development from being suppressed by BP [59,60,61,62,63,64,65].

## 6. New Perspectives about the Therapeutic Potential of Non-Coding RNAs

### 6.1. miR-29-3p Inhibitors

Even if the expression of the miR-29 family is present in osteoblast and osteoclast lineage, scientists demonstrated that a slight 30–60% drop in miR-29-3p family members had no effect on osteoclast quantity or function, using mice that were universally expressing a miR-29-3p tough decoy [41]. On the other hand, decoy expression reduced the amount of bone formed in growing mice, which in turn reduced the volume of trabecular bone in mature animals [42]. These findings corroborate earlier in vitro research indicating that miR-29-3p functions as an osteoblast differentiation positive regulator and, compared to wild type, cortical thickness, osteoblast surface, bone formation rate, and Ctnnb1/β-catenin mRNA—a miR-29-3p target—were all increased in miR-29 decoy animals treated with intermittent parathyroid hormone (PTH). These results point to distinctions between the processes governing bone development at the basal level and bone formation brought on by sporadic PTH. Using osteoblastic cell lines, in vitro experiments showed that miR-29-3p is a positive regulator of osteoblastogenesis, specifically targeting multiple Wnt signaling antagonists, Hdac4 and Tgfb3 as negative regulators of osteoblastic differentiation [45]. Interestingly, the miR-29 family is also well known for its capacity to target mRNAs specific to the extracellular matrix, such as those encoding type I collagens and non-collagenous extracellular matrix components that control the formation of fibrils, mineralization, and the activity of growth factors [41]. In vitro, early osteoblasts—which deposit a significant amount of extracellular matrix—have low levels of miR-29-3p; then, as the matrix ages and turns mineralized, the expression of the miR-29-3p family members rises. According to published data [41], the cell modifies the levels of miR-29 to facilitate the gene expression programs required at various stages of the osteoblastic differentiation program. Interestingly, it has been demonstrated in vitro that PTH therapy could induce osteocytes to return to a less differentiated phenotype [45]. Moreover, intermittent PTH did not significantly regulate miR-29-3p isoforms in bone in wild-type mice, while reduced miR-29-3p levels might have encouraged the phenotype linked to PTH therapy that is “less differentiated”, because of the decoy’s expression [42]. Rankl was later found to be a novel target of miR-29-3p, which adds to an indirect way in which miR-29-3p controls the quantity of osteoclasts in vivo [41]. The miR-29-3p decoy mice’s bone did not show a substantial rise in Rankl mRNA, but osteoprotegerin (Opg) mRNA was reduced, leading to an enhanced Rankl/Opg ratio that may encourage the production of osteoclasts [42]. Considering a decrease in miR-29-3p activity in osteoclasts themselves, this could be seen as a possible compensatory reaction by the osteoblast lineage to preserve skeletal homeostasis by boosting osteoclast quantity and function. Finally, it has been demonstrated that intermittent PTH increased cortical thickness more than in wild-type mice and that BCL2 modifying factor, an apoptosis activator, is a novel miR-29-3p target capable of inhibiting it using a tough decoy [41]. These findings may support the co-administration of PTH and miR-29-3p inhibitors to promote bone formation, particularly in the cortical compartment [42].

### 6.2. Targeting Maternally Expressed Gene 3 to Promote Osteogenesis

The 14q32.2 Maternally expressed gene 3 (MEG3) lncRNA is believed to function as a tumor suppressor via both p53-dependent and p53-independent mechanisms, and its expression is epigenetically regulated. Benetatos and colleagues studied the expression of MEG3 in multiple myeloma and found that, in approximately 60% of patients, the differentially methylated region (DMR) of the MEG3 promoter was hypermethylated [45]. This correlated with both the presence of bone disease and the stage. It is interesting to note that during osteogenic development, mesenchymal stem cells from MM patients expressed MEG3 less than those from normal donors and that MEG3 overexpression was able to induce differentiation [46]. In fact, different experiments showed that MEG3 is capable of stimulating osteogenesis by interfering with the transcription of the Transforming Growth Factor (TGF) family member gene BMP4 [47]. Moreover, MEG3 will directly interact with the transcription factor SOX2, causing it to dissociate from the BMP4 promoter and thus leading to an upregulation of BMP4 expression [15].

### 6.3. OIP5-AS1 Blocks the Pro-Oncogenic miR-410

Yang and colleagues found an unfavorable relationship between the long non-coding RNA OIP5 antisense RNA 1 (lncRNA OIP5-AS1), located on chromosome 15q15.1, and the high expression levels of miR-410 in newly diagnosed multiple myeloma [66]. Empirical data demonstrated that OIP5-AS1 may negatively regulate the expression of miR-410 and its pro-tumor effects on NCI-H929 and RPMI-8226 plasmacytoma cells’ cell division, cell cycle progression, and apoptosis [67]. It was demonstrated that in cell lines from newly diagnosed multiple myeloma and relapsed cases, miR-410 expression was elevated, and this fact has been shown to be correlated with in vitro and in vivo cell proliferation, cell cycle advancement, and prevention of apoptosis. Furthermore, it was discovered that KLF10 was a direct target of miR-410, mediating the influence of miR-410 that led to PTEN/AKT activation. The biological effects of miR-410 on multiple myeloma cells were at least partially eliminated by altering KLF10 expression or using an AKT inhibitor. Additionally, there was an inverse correlation between the level of miR-410 and the downregulated expression of lncRNA OIP5-AS1, which has the ability to control the expression of miR-410 and the cellular activities mediated by KLF10/PTEN/AKT that it targets [15].

## 7. Discussion

**Figure 3:**
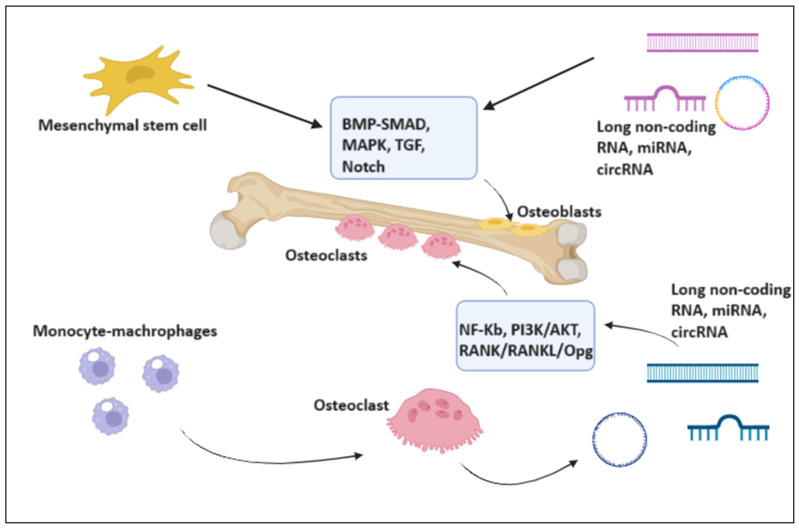
miRNA, lncRNA and circRNA affect osteoclasts, participating in the development of ONJ. Created with BioRender.com (accessed on 19 November 2023).

When antiresorptive and antiangiogenic drugs are used without preceding radiation therapy, patients may develop osteonecrosis of the jaw [68,69,70,71]. The inflammatory pathway of receptor activator of nuclear factor NF-kB ligand and macrophage colony-stimulating factor are involved in the pathogenesis of this condition. These factors are necessary for the survival and proliferation of osteoclast precursors; in fact, they signal the cell via their receptor c-Fms, which in turn activates extracellular signal-regulated kinase and phosphoinositide 3-kinase/Akt [72,73,74,75,76,77,78,79]. Non-coding RNAs have been implicated in the pathophysiology of mandibular osteonecrosis, and this information is helpful for diagnosis because these small RANs may be biomarkers of apoptotic activity in bone. It has also been demonstrated that miR-29 and miR-31-5p, which act on specific targets like RhoA and CALCR, promote osteonecrosis through programmed cell death [80,81,82,83,84]. It was discovered that the expression of miR-29, miR-31-5p, hsa-miR-422a, hsa-miR-148a-3p, miR-183-5p, miR-214-3p, and miR-9718 encouraged osteoclastogenesis [15]. On the other hand, it has been demonstrated that the expression of the following miRNAs impeded the process of osteoclastogenesis: miR-7b-5p, miR-26a-5p, miR-34a-5p, miR-124-3p, miR-125a-5p, miR-146a-5p, and miR-218-5p [15,24]. Moreover, it was shown that three miRNAs, hsa-miR-133a-3p, miR-21-5p, and miR-223-3p, had both promoting and inhibitory effects [6]. A positive regulator of osteoclast formation, miR-29 (a/b/c) family expression was upregulated during osteoclastogenesis in vitro cell culture [33]. This was consistent with the expression of TRAP and cathepsin K, two markers of osteoclasts [34,35,36,37]. The overexpression of miR-218-5p, a negative regulator of osteoclastogenesis, was associated with the decreased expression of TRAP and Cathepsin K [15,34]. Conversely, it has been demonstrated that some long non-coding RNAs are expressed at lower amounts in patients with osteonecrosis and multiple myeloma. These RNAs are linked to the inhibition of osteoblast development, which affects the mandibles as lesions advance. Recent research evaluated the function of long noncoding RNAs (lncRNAs) during osteogenic lineage commitment or osteocyte terminal differentiation. For example, during osteogenesis, lncRNA-1 is expressed more [48]. Furthermore, there is proof that circular RNAs can lessen the inhibitory effect of bisphosphonates on the process of osteoclastogenesis [56]. In the absence of BP medication, researchers assessed the effect of the mmu_circ_0001066 inhibitor on RANKL-induced osteoclastogenesis [58,85,86]. TRAP staining and activity showed a significant reduction in osteoclast differentiation caused by the mmu_circ_0001066 inhibitor. These results highlight that overexpression of mmu_circ_0001066 prevented BP from suppressing the osteoclast growth of RAW264.7 cells [87,88,89,90,91,92,93,94,95,96,97,98,99,100,101,102] (Figure 3).

## 8. Conclusions

The progressive loss and destruction of bone typical of osteonecrosis of the jaw is a crucial problem in the management of patients with multiple myeloma. All the data presented clearly show the role of non-coding RNA in the pathogenesis of the disease in patients with multiple myeloma treated with bisphosphonates and their possible use as biomarkers at the diagnosis and prognosis phases.

While for lnc-RNAs and circ-RNAs there is only correlative evidence with onset and progression of the osteonecrosis of the jaw, miRNAs showed in vivo to have functionality; in detail, an overexpression of miR-16-1, miR-21, miR-23a, miR-28, miR-101-1, miR-124-1, miR-129, miR-139, miR-145, miR-149, miR-202, miR-221, miR-424, and miR-520 has been detected in patients with multiple myeloma and osteonecrosis compared to controls.

It would be interesting to find strategies to overcome the loss of bone in osteonecrosis of the jaw and stimulate the formation of new tissue. Support might come from Maternally expressed gene 3 to promote osteogenesis, since it has been demonstrated it interferes with the transcription of the TGF family member gene BMP4 and interacts with the transcription factor SOX2, stimulating osteogenesis.

## Figures and Tables

**Table 1:** miRNAs and related target genes acting on osteoclasts.

miRNA	Target Genes	Over/Down Expression	Effect on Apoptosis
miR-29	SRGAP2, NFIA, CD93, CALCR	Over	Promotion
miR-31-5p	*RhoA*	Over	Promotion
miR-183-5p	*HO-1*	Over	Promotion
miR-7b-5p	DC-STAMP	Down	Inhibition
miR-125a-5p	TRAF6	Down	Inhibition
miR-503-5p	RANK	Down	Inhibition
miR-214-3p	Pten	Over	Promotion
